# Advantages and Disadvantages of Random Forest Models for Prediction of Hip Fracture Risk Versus Mortality Risk in the Oldest Old

**DOI:** 10.1002/jbm4.10757

**Published:** 2023-07-03

**Authors:** Lisa Langsetmo, John T. Schousboe, Brent C. Taylor, Jane A. Cauley, Howard A. Fink, Peggy M. Cawthon, Deborah M. Kado, Kristine E. Ensrud, for the Osteoporotic Fractures in Men (MrOS) Research Group

**Affiliations:** ^1^ Center for Care Delivery and Outcomes Research, VA Health Care System Minneapolis MN USA; ^2^ Department of Medicine University of Minnesota Minneapolis MN USA; ^3^ Rheumatology Research HealthPartners Institute Bloomington MN USA; ^4^ Division of Health Policy & Management, School of Public Health University of Minnesota Minneapolis MN USA; ^5^ Division of Epidemiology & Community Health, School of Public Health University of Minnesota Minneapolis MN USA; ^6^ Department of Epidemiology, School of Public Health University of Pittsburgh Pittsburgh PA USA; ^7^ Geriatric Research Education and Clinical Center, VA Health Care System Minneapolis MN USA; ^8^ California Pacific Medical Center Research Institute San Francisco CA USA; ^9^ Department of Medicine Stanford University Stanford CA USA; ^10^ Geriatric Research Education and Clinical Center, VA Health Care System Palo Alto CA USA

**Keywords:** HIP FRACTURE, MACHINE LEARNING, RANDOM FOREST

## Abstract

Targeted fracture prevention strategies among late‐life adults should balance fracture risk versus competing mortality risk. Models have previously been constructed using Fine‐Gray subdistribution methods. We used a machine learning method adapted for competing risk survival time to evaluate candidate risk factors and create models for hip fractures and competing mortality among men and women aged 80 years and older using data from three prospective cohorts (Study of Osteoporotic Fractures [SOF], Osteoporotic Fracture in Men study [MrOS], Health Aging and Body Composition study [HABC]). Random forest competing risk models were used to estimate absolute 5‐year risk of hip fracture and absolute 5‐year risk of competing mortality (excluding post–hip fracture deaths). Models were constructed for both outcomes simultaneously; minimal depth was used to rank and select variables for smaller models. Outcome specific models were constructed; variable importance was used to rank and select variables for inclusion in smaller random forest models. Random forest models were compared to simple Fine‐Gray models with six variables selected a priori. Top variables for competing risk random forests were frailty and related components in men while top variables were age, bone mineral density (BMD) (total hip, femoral neck), and frailty components in women. In both men and women, outcome specific rankings strongly favored BMD variables for hip fracture prediction while frailty and components were strongly associated with competing mortality. Model discrimination for random forest models varied from 0.65 for mortality in women to 0.81 for hip fracture in men and depended on model choice and variables included. Random models performed slightly better than simple Fine‐Gray model for prediction of competing mortality, but similarly for prediction of hip fractures. Random forests can be used to estimate risk of hip fracture and competing mortality among the oldest old. Modest gains in performance for mortality without hip fracture compared to Fine‐Gray models must be weighed against increased complexity. © 2023 The Authors. *JBMR Plus* published by Wiley Periodicals LLC on behalf of American Society for Bone and Mineral Research. This article has been contributed to by U.S. Government employees and their work is in the public domain in the USA.

## Introduction

Targeted fracture prevention strategies among late‐life adults should take into account the competing risk of mortality as it is materially related to both absolute and relative fracture risks.^(^ ^1^, ^2^, ^3^ ^)^ Although there has been substantial research investigating the risk factors for hip fracture^(^ ^4^, ^5^, ^6^, ^7^ ^)^ and for mortality post–hip fracture,^(^ ^8^, ^9^, ^10^, ^11^ ^)^ very little research has been devoted to estimating the varying magnitude of hip fracture risk versus the competing risk of mortality without hip fracture among the oldest old. Using a simple five‐variable model estimated using the Fine‐Gray approach,^(^ ^12^ ^)^ a method derived from survival time which accounts for the competing risk, we have shown that fracture and competing mortality have different risk factors in older men leading to different risk profiles.^(^ ^13^ ^)^ We sought to extend and improve this prior work by considering additional risk factors in a larger and more diverse cohort of late‐life adults.

The choice of the modeling approach represents one potential area of improvement in methodology used to address fracture prediction in late‐life. Many studies have considered machine learning methods applied to fracture risk prediction by considering fracture as a binary outcome, which presupposes either cross‐sectional data or complete and fixed follow‐up.^(^ ^14^, ^15^, ^16^, ^17^ ^)^ In contrast, there are only a few machine learning methods that have been developed for use with survival time data with competing risks. Random forests were first introduced by Breiman^(^ ^18^ ^)^ as an extension of classification and regression trees and have subsequently been adapted for use with survival time data and predictions in the presence of competing risks.^(^ ^19^ ^)^ Random forests are by their nature nonparametric. Thus, they are extremely flexible and make few assumptions about the relationships between variables.

Our primary aim was to use random forest models to select important risk factors for the 5‐year absolute risk of hip fracture accounting for the competing risk of mortality and the 5‐year absolute risk of mortality (excluding post hip fracture death) among men and women age 80 years and older. We evaluated model performance and created simple Fine Gray models for the purpose of comparison. Our secondary aim was to assess the advantages and disadvantages of a random forest models compared to Fine Gray models to generate risk predictions for fracture and competing mortality.

## Subjects and Methods

### Participants

We studied community‐dwelling participants enrolled in three prospective cohort studies of older adults: Study of Osteoporotic Fractures (SOF),^(^ ^4^ ^)^ Osteoporotic Fracture in Men study (MrOS),^(^ ^20^, ^21^ ^)^ and Health Aging and Body Composition study (HABC). Further details on study site and eligibility are shown in Table S1. SOF enrolled 9704 community‐dwelling white women 65 years and older at four clinical centers in 1986–1988 with expansion of the total cohort to 10,366 women with the addition of 662 black women 65 years and older at Year 10 (1997–1998). MrOS enrolled 5994 community‐dwelling men 65 years and older at six clinical centers in 2000–2002. Health ABC enrolled 3075 black and white community‐dwelling older adults age 70 to 79 years with no self‐report of mobility difficulty at two clinical centers at the baseline exam (1997–1998). Participants were eligible for the current study if they were at least 80 years old with measurement of BMD at a clinic examination (SOF Year 10 or 16, MrOS Year 4.5, 7, 14, and Health ABC Year 3, 5, 6, 8 or 10). Participants were only included once and entered into the analysis at the examination when they first reached at least age 80 years (index examination) (Table S2).

### Ascertainment of outcomes of hip fracture and mortality without hip fracture

SOF and MrOS participants were queried every 4 months and HABC participants every 6 months to ascertain vital status and incident fractures, including hip fractures. The response rate for these contacts was over 95% for active surviving participants in all cohorts. Self‐reported hip fractures in all cohorts were confirmed by radiographic reports. Deaths were verified with death certificates. Participant follow‐up was included until event (hip fracture, death) or censoring (maximum 5 years after the index examination). The mean ± SD follow‐up time to the first occurrence of hip fracture, mortality or censoring was 4.4 ± 1.2 years for both men and women.

### Candidate risk factors

Age, self‐reported race/ethnicity, recalled height and weight at age 25 years, and history of fracture after age 50 years were collected at baseline in each cohort. Height, weight, health status, and smoking status were recorded at each examination. Long‐term height loss was calculated as the difference between measured height at the index examination and height at age 25 years, whereas current measured weight at index examination expressed as percentage of self‐reported weight at age 25 years indicated direction and magnitude of long‐term weight change. History of fracture at the index examination was determined based on history of fracture since age 50 years at the baseline exam and confirmed fractures between the baseline and index examination and categorized as follows: recent (within last 5 years), not recent but after age 50 years, and no fracture after age 50 years. Fall history in the previous year was assessed using triannual postcards.

Clinic measures at the index examination included components of phenotypic frailty; slowness (walk speed <0.6 m/s in women, <0.8 m/s in men), weakness (grip strength <20 kg in women, <32 kg in men), poor energy, low physical activity, and shrinking.^(^ ^22^ ^)^ Usual walk speed was measured over a 6‐m course in SOF and MrOS and a 4‐m course in HABC with the latter recalibrated to match 6‐m gait speed^(^ ^23^ ^)^ and grip strength was measured using a hand‐held dynamometer. Those unable to perform the walk speed or grip strength tests were assigned a value of 0 for the continuous measure. Shrinking was defined as recent weight loss >10 lbs or >5% of baseline weight or current weight <18.5 kg/m^2^. Participants also self‐reported if they had poor energy and low physical activity (defined as not walking for exercise and not engaging in at least moderate physical activity). Health status at the index examination was self‐reported as excellent, good, fair, poor, or very poor. Time required to stand from a chair five times without using the arms was measured and converted to a ratio of stands per second; weakness (chair stands) was indicated by inability to perform the test. At the index examination, participants self‐reported their ability to walk two to three blocks on level ground or climb 10 stairs without difficulty (scored 0), with some difficulty (scored 1), with a lot of difficulty (score 2), or unable (score 3). The scores for these two mobility tasks were combined into one variable, ranging from 0 to 6.

Participants were asked to bring all current prescription medication containers with them to the clinic. Medications were recorded in an electronic medication inventory database and matched to its ingredients(s) based on the Iowa Drug information Service drug vocabulary (College Pharmacy, University of Iowa, Iowa City, IA, USA).^(^ ^24^ ^)^ We considered each medication class as a candidate predictor variable of incident hip fracture and mortality without hip fracture based on prior medical literature review and a biologically plausible mechanism by which each medication class may be associated with risk of hip fracture and/or mortality. Candidate medications included: antidepressants, medications for type 2 diabetes, anti‐epileptic medications, angiotensin converting enzyme (ACE) inhibitors, benzodiazepines, beta blockers, angiotensin receptor blockers (ARB), systemic corticosteroids, calcium channel blockers, H1 receptor antagonists, H2 receptor antagonists, loop diuretics, potassium sparing diuretics, thiazide diuretics, narcotics, proton pump inhibitors, thyroid hormone replacement therapy, selective serotonin receptor inhibitors (SSRIs), and warfarin.

Participants were asked if they had been diagnosed by a health care provider as having arthritis, breast cancer (women), prostate cancer (men), cancer, coronary heart disease (angina or myocardial infarction), stroke, heart failure, diabetes, dementia, depression, and Parkinson's disease. Participants were considered to have diabetes, Parkinson's disease, depression, or dementia if they either self‐reported a physician diagnosis or were treated with medication used to treat that diagnosis.

BMD at the total hip was measured at the index examination with dual‐energy X‐ray absorptiometry (DXA; QDR 4500 W; Hologic, Inc., Bedford, MA, USA) using standardized protocols as described.^(^ ^25^, ^26^, ^27^ ^)^ Extensive quality control procedures were carried out at all examinations including centralized training and certification of DXA technicians and scanning of a central hip phantom at each clinical center at regular intervals.

### Statistical analysis

All analyses were stratified by sex. The survival data was truncated at 5 years with prediction and outcome calculated at that time‐point. We used random survival forest models (RSFs), a machine learning approach that uses tree‐based models to create survival‐time predictions accounting for competing risk outcomes (hip fracture and mortality without hip fracture).^(^ ^19^ ^)^ RSF does not assume linearity or functional form and allows for complex interactions between variables. RSF has several component steps: Step 1: draw bootstrap samples, Step 2: construct tree for each bootstrap sample using recursive partitioning based on randomly selected variables and cut‐points, Step 3: average model estimates over all trees to obtain (forest) estimate for full data. There were two types of forests depending on the splitting rule in Step 2. To construct a single forest for BOTH outcomes (hip fracture and mortality without hip fracture), the splitting rule was based on a modified log‐rank test adapted for competing risks.^(^ ^28^ ^)^ To construct outcome specific forests, the splitting rule was based on a log‐rank test for the chosen outcome (hip fracture or mortality without hip fracture). Missing values were present for some variables. In order to keep all variables under consideration without limiting the sample to those participants with complete data for all candidate risk factors, we used random forest imputation prior to model construction.^(^ ^29^ ^)^

Variable selection for random forests modeling both outcomes was based on minimal depth (MD)^(^ ^30^ ^)^ because this measure is both stable and takes both outcomes into account, while selection for outcome specific forests was based on variable importance (VIMP)^(^ ^18^ ^)^ to optimize outcome specific discrimination. Minimal depth for a variable is found by taking distance from the root of a tree to the maximal subtree with the given variable as the base node. Variables with low minimal depth are those that better predict outcome status (both hip fracture and mortality before hip fracture). Permutation VIMP compares the estimated error from sample data to that where the given variable has been replaced by a new variable which is a random permutation of the original variable. By construction, higher VIMP indicates greater impact of the given variable on estimated model error compared to replacement with randomly permuted (noninformative) variable, and thus greater variable importance.

Model performance was assessed using out‐of‐bootstrap sample estimates, ie, model performance corrected for optimism. Each random forest depends on the bootstrap sample selection and estimated VIMP depends on permutations. Thus, the set of variables chosen for the final model differ slightly across different bootstrap samples. Sensitivity analyses were performed to assess dependence of variable selection and model performance on random seeds. We created parsimonious outcome specific models by dropping all variables with VIMP <0.003 in the given model run.

Finally, we created simple Fine‐Gray models^(^ ^12^ ^)^ for the outcomes of hip fracture and mortality without hip fracture based on six selected predictors. For simplicity, we started with our previously published simple model based on five independent variables: age, femoral neck BMD, prior fracture, fall history, and number of chronic medical conditions.^(^ ^13^ ^)^ We added one additional independent variable to this model, the number of frailty phenotype components, because it was a single variable summary of many important predictors in the random forest models. To keep the Fine‐Gray models parsimonious, we did not consider higher order terms or interactions. We also assumed constant subdistribution hazard ratios for ordinal variables.

Analysis was performed using Stata 17 and R (v 4.2.2) using the R‐packages randomForestSRC^(^ ^31^ ^)^ (version 3.1.1) and ggRandomForest, version 2.2.1. Analytic details and sample code provided in the Supporting Information.

### Role of the funding source

The funding agencies had no direct role in the conduct of the study; the collection, management, analyses and interpretation of the data; or preparation or approval of the manuscript.

## Results

The characteristics at the index examination of the 3989 men and 4953 women included in the analyses are shown in Table 1. Among the men, the average age was 82.7 years; mean weight was 79.4 kg and mean femoral neck BMD *T*‐score was 0.8 (using young female reference data). Of these men, 46.5% had at least two chronic conditions, 16.4% reported two or more falls in the last year, 8% experienced a fracture within the past 5 years, and 18.1% had long‐term height loss of at least 8 cm. Among the women, the average age was 82.6 years; mean weight was 65.4 kg and mean femoral neck BMD *T*‐score was −1.0. Of these women, 41.1% had at least two chronic conditions, 33.7% reported two or more falls in the last year, 15.1% experienced a fracture within the past 5 years, and 21% had long‐term height loss of at least 8 cm. During the 5‐year follow‐up, 123 (3.1%) men and 325 (6.6%) women had an incident hip fracture; 922 (23.1%) men and 824 (16.6%) women died without experiencing an incident hip fracture.

**Table 1:** Baseline Characteristics Stratified by Sex

Predictor variables	Men (*n* = 3989)	Women (*n* = 4953)
Age (years), mean ± SD	82.7 ± 2.7	82.6 ± 2.7
Height (cm), mean ± SD	172.1 ± 6.7	156.9 ± 6.1
Weight (kg), mean ± SD	79.4 ± 12.7	65.4 ± 12.7
Current weight as % age 25 weight, mean ± SD	111 ± 17	117 ± 21
Number of frailty components (0 to 5), mean ± SD	1.8 ± 1.3	2.5 ± 1.3
Number of medications (0 to 20, truncated), mean ± SD	7.8 ± 4.5	6.6 ± 3.8
Grip strength (kg), mean ± SD	35.3 ± 9.7	18.4 ± 5.8
Gait speed (m/s), mean ± SD	1.07 ± 0.24	0.85 ± 0.26
Chair stand speed (#/s), mean ± SD	0.37 ± 0.18	0.32 ± 0.19
Total hip BMD (g/cm^2^), mean ± SD	0.929 ± 0.148	0.727 ± 0.141
Femoral neck BMD (g/cm^2^), mean ± SD	0.756 ± 0.134	0.630 ± 0.125
Femoral neck BMD *T*‐score, mean ± SD	−0.8 ± 1.1	−1.9 ± 1.0
Race/ethnicity, *n* (%)		
White	3478 (87.2)	4402 (88.9)
Black/Other	511 (12.8)	551 (11.1)
Number of chronic conditions, *n* (%)		
None	868 (21.8)	1268 (25.6)
1	1266 (31.7)	1651 (33.3)
2+	1855 (46.5)	2034 (41.1)
Mobility score (0 to 6), *n* (%)		
0	2986 (75.5)	3104 (63.8)
1	440 (11.1)	612 (12.6)
2+	528 (13.4)	1153 (23.7)
Fall history (number of falls in 12 months), *n* (%)		
None	2632 (66.0)	3272 (66.3)
1	703 (17.6)	968 (19.6)
2+	654 (16.4)	697 (14.1)
Fracture history, *n* (%)		
None after age 50 years	2854 (71.5)	2423 (50.4)
After 50 years, >5 years ago	814 (20.4)	1654 (34.4)
Recent (≤5 years ago)	321 (8.0)	727 (15.1)
Smoking history, *n* (%)		
Never	1595 (40.1)	3087 (62.7)
Former	2312 (58.1)	1683 (34.2)
Current	74 (1.9)	152 (3.1)
Height loss since age 25 years, *n* (%)		
<4 cm	1362 (34.2)	1,623 (33.0)
4–8 cm	1903 (47.7)	2260 (46.0)
8 cm or more	722 (18.1)	1032 (21.0)
Self‐reported health, *n* (%)		
Excellent	1032 (25.9)	967 (19.6)
Good	2077 (52.2)	2584 (52.4)
Fair/poor/very poor	869 (21.8)	1383 (28.0)
Incident events: (5 years), *n* (%)		
None	2944 (73.8)	3804 (76.8)
Hip fracture	123 (3.1)	325 (6.6)
Death without hip fracture	922 (23.1)	824 (16.6)

### Variable rankings

**Fig. 1:**
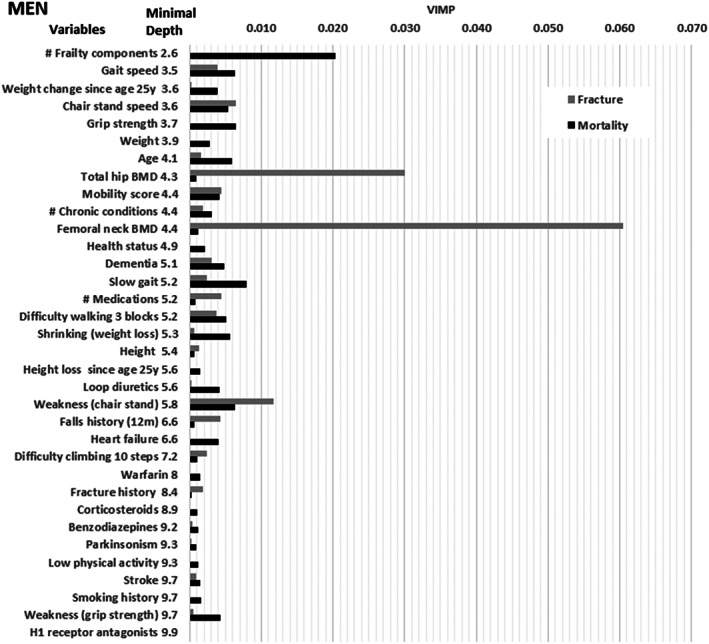
A comparison of minimal depth and variable importance (VIMP) for hip fracture and mortality before hip fracture among older men*. *Minimal depth obtained from model predicting both outcomes, VIMP obtained from outcome specific model. Variables with minimal depth > 10 dropped. Top 5 variables based on: (1) Minimal Depth: # Frailty components, gait speed, weight change since age 25 years, chair stand speed, grip strength). (2) Hip fracture VIMP: Femoral neck BMD, total hip BMD, weakness (chair stand), chair stand speed, # medications). (3) Mortality VIMP: # Frailty components, slow gait, grip strength, weakness (chair stand), gait speed).

**Fig. 2:**
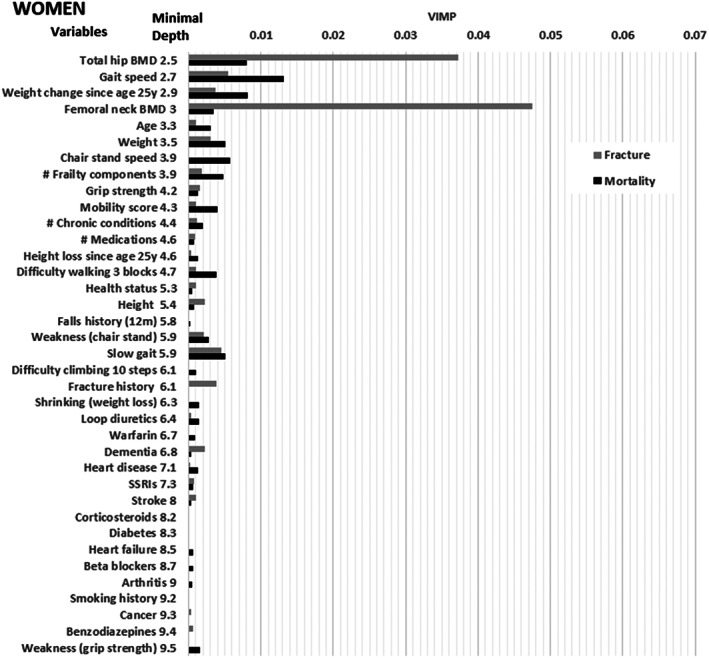
A Comparison of Minimal Depth and Variable Importance (VIMP) for Hip Fracture and Mortality before Hip Fracture Among Older Women*. *Minimal depth obtained from model predicting both outcomes, VIMP obtained from outcome specific model. Variables with minimal depth >9.5 dropped. Top 5 variables based on: (1) Minimal depth: Total hip BMD, gait speed, weight change since age 25 years, femoral neck BMD, age. (2) Hip fracture VIMP: Femoral neck BMD, total hip BMD, weight change since age 25 years, gait speed, weight. (3) Mortality VIMP: Gait speed, weight change since age 25 years, age, weakness (chair stand), difficulty walking 3 blocks.

Figures 1 and 2 show the MD from the full model predicting both outcomes and the variable importance for hip fracture derived from the full model predicting hip fracture model and the variable importance for mortality from the full model predicting mortality before hip fracture in men, respectively, women. Table S3 provides the associated ranking of variables from these models (better predictors have lower ranking). There are clear differences in rankings between men and women, and between MD rankings from the model considering both hip fracture and mortality and VIMP rankings from event specific models. Rankings were robust to random seed, i.e., there was a correlation of 0.99 between MD or VIMP on models with new random seed compared to original models leading to nearly identical rankings.

Among both men and women, gait speed and weight change since age 25 years were included in the list of the five variables with lowest MD based on a random forest designed to predict both hip fracture and mortality. Among men, the three additional variables with low minimal depth were number of frailty components, grip strength, and chair stand speed. In contrast, the three additional variables with the lowest MD in women were total hip BMD, femoral neck BMD and age.

Among both men and women, total hip BMD and femoral neck BMD were each included in the list of five variables with highest VIMP based on a random forest designed to predict hip fracture. Among men, the three additional variables with the highest VIMP for hip fracture prediction included inability to perform chair stand test, chair stand speed, and number of medications, while in women the three additional variables with the highest VIMP for hip fracture prediction included weight change since age 25 years, gait speed, and weight.

Among both men and women, gait speed and inability to do chair stands were included in the list of the five variables with highest VIMP based on a random forest designed to predict mortality before hip fracture. Among men, the three additional variables with the highest VIMP for mortality prediction were number of frailty components, grip strength, and slowness, whereas in women the three additional variables with the highest VIMP were age, weight change since age 25 years and difficulty walking three blocks.

### Model performance

The overall performance of the random forest models (full model including all candidate risk factors and more parsimonious models based on MD or VIMP selection criteria) is shown in Table 2. Model performance as measured by Harrell's C‐statistic depended upon sex, split criteria, and variable selection approach and varied from 0.65 (prediction of competing mortality from seven‐variable hip fracture model in women based on VIMP selection criteria) to 0.81 (prediction of hip fracture from 10‐variable hip fracture model in men based on VIMP selection criteria).

**Table 2:** Variable Selection and Model Performance for Specific Random Forest Models

Split rule outcome	Variable selection	Number of variables	Fracture C‐statistic	Mortality C‐statistic
Men				
Both	None	57	0.78	0.69
	Minimal depth	34	0.78	0.69
Fracture	None	57	0.79	0.69
	Variable importance	10	0.81	0.65
Mortality	None	57	0.77	0.70
	Variable importance	16	0.68	0.68
Women				
Both	None	58	0.74	0.68
	Minimal depth	37	0.75	0.68
Fracture	None	58	0.74	0.68
	Variable importance	7	0.73	0.65
Mortality	None	58	0.74	0.67
	Variable importance	12	0.73	0.67

All models were run with particular random seed. Minimal depth selected variables shown in Fig. 1 and Table S3. Outcome specific models were run with restricting to variables with variable importance (VIMP) > 0.003 as noted below.

Hip fracture in men (*n* = 10): femoral neck BMD, total hip BMD, weak (chair stands), chair stand speed, # medications, mobility score, fall history, gait speed, difficulty walking 3 blocks, dementia.

Mortality in men (*n* = 16): # frailty components, slow (gait speed), grip strength, weak (chair stands), gait speed, age, shrinking (weight loss), chair stand speed, difficulty walking 3 blocks, dementia, weak (grip strength), mobility score, loop diuretic use, heart failure, % of weight at age 25 years, # chronic conditions.

Hip fracture in women (*n* = 7): femoral neck BMD, total hip BMD, % of weight at age 25 years, gait speed, weight, slow (gait speed), fracture history.

Mortality in women (*n* = 12): gait speed, % of weight at age 25 years, age, weak (chair stands), difficulty walking 3 blocks, weight, total hip BMD, mobility score, coronary heart disease, # frailty components, chair stand speed, loop diuretic use.

The first set of random forest models was designed to predict both hip fracture and mortality without hip fracture using modified log‐rank test to split nodes. For the models including nearly 60 variables (57 in men and 58 in women), the discrimination as determined by the C‐statistic was higher for hip fracture outcomes (0.78 in men, 0.74 in women) than for competing mortality (0.69 in men, 0.68 in women). Using the same criteria to split nodes, models with fewer predictors were obtained by dropping variables with high minimal depth; discrimination of these models with fewer independent variables (34 in men, 37 in women) was similar to the larger models.

The next set of random forest models focused on predicting incident hip fracture. The model with the full set of predictors had similar discrimination for both hip fracture and mortality before hip fracture outcomes to the first series of models predicting both outcomes. Selecting only variables with fracture VIMP >0.003, it was possible to achieve nearly the same discrimination for hip fracture with only 10 variables in men and seven variables in women, although the discrimination for mortality without fracture was 0.03–0.04 lower for the smaller models than it was for the larger models.

The final set of random forest models focused on predicting mortality without hip fracture. The model with the full set of predictors had similar discrimination for both hip fracture and competing mortality outcomes to the first series of models predicting both outcomes.

By selecting only variables with mortality VIMP >0.003, it was possible to obtain nearly the same discrimination for competing mortality with only 16 variables in men and 12 variables in women. The fracture discrimination was substantially lower (by 0.09) for the parsimonious model compared to the larger model in men, but remained similar in women. We note that BMD variables were not included in the smaller model for men, but total hip BMD remained in the smaller model for women.

### Comparison Fine‐Gray model

Table 3 shows subdistribution hazards (SHR) from a six‐variable comparison model constructed using the Fine‐Gray method. Among men, older age (SHR = 1.34, 95% confidence interval [CI]: 1.00–1.80 per 5 years increase), lower femoral neck BMD (SHR = 2.87, 95% CI: 2.26–3.64 per 1 point decrease in *T*‐score), and a higher number of falls in the past year (SHR = 1.23, 95% CI: 1.05–1.44 per fall) were associated with increased risk of hip fracture. Among women, lower femoral neck BMD (SHR = 2.26, 95% CI: 1.96, 2.60 per 1 point decrease in *T*‐score) and recent fracture (SHR = 1.55, 95% CI: 1.15, 2.09 recent versus none after age 50 years) were associated with increased risk of hip fracture. Among both men and women, older age, greater number of chronic conditions, greater number of frailty components were associated with increased mortality without hip fracture, and among women lower BMD was also associated with increased mortality without hip fracture.

**Table 3:** The Association Between Selected Risk Factors and 5‐Year Risk of Hip Fracture Based on Simple Competing Risk Model in Men and Women

	Hip fracture		Mortality	
Parameter	SHR	95% CI	SHR	95% CI
Men (*n* = 3989) 123 hip fractures, 922 deaths				
Age (per 5 year increase)	**1.34**	**(1.00, 1.80)**	**1.35**	**(1.20, 1.51)**
Femoral neck BMD (per 1 SD decrease)	**2.87**	**(2.26, 3.64)**	1.05	(0.98, 1.12)
Fracture history				
None after age 50 years	Ref		Ref	
After 50 years, >5 years ago	1.13	(0.74, 1.74)	0.90	(0.76, 1.06)
Recent (≤5 years ago)	1.43	(0.85, 2.38)	1.07	(0.86, 1.33)
Number of falls in last 12 months (per fall)	**1.23**	**(1.05, 1.44)**	1.00	(0.94, 1.07)
Number of chronic conditions (per condition)	1.12	(0.98, 1.29)	**1.18**	**(1.12, 1.24)**
Number of frailty components (per component)	1.03	(0.88, 1.19)	**1.39**	**(1.31, 1.47)**
C‐statistic for full model	0.80	95% CI (0.76, 0.83)	0.67	95% CI (0.65, 0.68)
Women (*n* = 4953) 325 hip fractures, 824 deaths				
Age (per 5 year increase)	1.12	(0.94, 1.34)	**1.54**	**(1.39, 1.71)**
Femoral neck BMD (per 1 SD decrease)	**2.26**	**(1.96, 2.60)**	**1.11**	**(1.03, 1.20)**
Fracture history				
None after age 50 years	Ref		Ref	
After 50 years, >5 years ago	**1.13**	**(0.87, 1.46)**	1.08	(0.93, 1.26)
Recent (≤5 years ago)	**1.55**	**(1.15, 2.09)**	1.02	(0.84, 1.25)
Number of falls in last 12 m (per fall)	1.01	(0.91, 1.12)	1.03	(0.96, 1.09)
Number of chronic conditions (per condition)	1.09	(0.99, 1.20)	**1.17**	**(1.11, 1.24)**
Number of frailty components (per component)	1.04	(0.94, 1.14)	**1.23**	**(1.16, 1.30)**
C‐statistic for full model	0.73	95% CI (0.71, 0.76)	0.65	95% CI (0.63, 0.67)

The random forest models with all predictors based on predicting both outcomes offered a slight improvement in the point estimate for the discrimination of mortality before hip fracture for both men (C‐statistic 0.69 versus 0.67) and women (C‐statistic 0.68 versus 0.65) compared with the simple Fine‐Gray models. In contrast, the random forest models offered no clear advantage for the discrimination of hip fracture compared with the simple Fine‐Gray models. In fact, the performance of the Fine‐Gray models for the prediction of hip fracture was comparable to the most parsimonious random forest models in both men and women albeit with even fewer total predictors (6 versus 7–10).

## Discussion

Random forest models with competing risks can be used to predict hip fracture and mortality without hip fracture among the oldest old. The main findings of the variable selection did not yield novel strong predictors for these outcomes, but rather reinforced the notion that hip fracture risk models are driven in large part by information from BMD variables, while competing mortality models are driven in large part by information related to markers or components of phenotypic frailty. Importantly, our findings indicate that most clinical risk factors aside from these main constructs have a modest impact on model performance (VIMP <0.005). While one might presume that there is a best model, results from our analyses suggest that models with very different sets of variables, including those with fewer variables, have very similar performance. Finally, while the point estimate of performance of the random forest models for mortality without hip fracture was in some cases better than the simple Fine‐Gray model, a salient question is whether possible improvement in model performance outweighs increase in model complexity. In the case of hip fracture accounting for competing risk of mortality, the Fine‐Gray model has similar performance to the random forest model while being a more parsimonious model. Parsimony enables better validation and testing of the model using external data and enhances feasibility of model for clinical use.

BMD is known to be a strong and consistent predictor of fracture. In our analysis, both total hip and femoral neck BMD are high‐ranking variables as determined by minimal depth and hip fracture VIMP criteria in men and women. However, in both men and women, total hip BMD is ranked higher by minimal depth criterion while femoral neck is ranked higher by hip fracture VIMP. This suggests that total hip BMD is favored when a simple binary predictor is needed because lower minimal depth reflects higher likelihood of variable with randomly selected binary cut‐point to be selected at a node, whereas femoral neck BMD is favored for overall model performance of the continuous variable, because VIMP measures error introduced by permutation of continuous variables. The clinical relevance of this finding is uncertain as it applies to randomly selected cut‐points, while clinical cut‐points tend to be fixed (eg, *T*‐score = −2.5).

Frailty as defined by phenotypic criteria is known to be a risk factor for both all‐cause^(^ ^32^ ^)^ and cause‐specific^(^ ^33^ ^)^ mortality. Among older women, phenotypic frailty has been shown to be associated with the constellation of falls, hip fracture and mortality.^(^ ^34^, ^35^ ^)^ The assessment of frailty as a risk factor for mortality does not directly address how frailty is related to mortality without hip fracture as the latter might well depend on the specific competing event. In the present study, certain components of the frailty phenotype (gait speed and weakness) had high variable importance for mortality without hip fracture among women, while the total number of frailty components had high variable importance for both hip fracture and mortality without hip fracture.

Very few binary variables were rated among the top predictors by any of the rankings. The notable exceptions were inability to perform chairs stands (men) and slowness defined by gait speed <0.6 m/s (women). Although neither measure is part of FRAX^(^ ^5^ ^)^ or Garvan^(^ ^7^ ^)^ fracture risk calculators, previous research has noted the relationship between poor physical performance and higher risk of hip fracture in both men^(^ ^36^ ^)^ and women.^(^ ^37^ ^)^ In contrast, we note that race/ethnicity (non‐Hispanic white versus black/other) was not chosen in any of our random forest models. Large population‐based samples have shown variation in hip fracture risk by race/ethnicity.^(^ ^38^ ^)^ Our analysis suggests that while there may be some association between this variable and fracture risk it has limited impact on overall model discrimination because other variables were consistently chosen in preference to race/ethnicity. Likewise, few medication classes or specific diseases were included as risk factors for hip fracture in our models. This finding may reflect that some of the fracture risk attributable to these medications is related to falls and fall history. We note that variable selection based on lower MD favors variables with more cut‐points while variable selection based on higher VIMP measures overall change in permutation model error which again may be very small due to prevalence of the risk factor. We also note that variable ranking does not reflect formal statistical tests and thus interpretation should be made accordingly.

Our approach to the machine learning was largely driven by the study question, in particular survival time and the relevance of the competing risk of mortality among the oldest old. Others have used a wide spectrum of machine learning approaches that consider hip fracture risk a classification problem, ie, they aim to create a classifier that best separates those who have hip fracture versus those who do not, a slightly different question amenable to several machine learning approaches. Su and colleagues^(^ ^39^ ^)^ used classification and regression tree analysis in the MrOS cohort and found that specific age and BMD cutoffs were associated with high hip fracture risk. Because our focus was strictly on adults age of 80 years and older, it is not surprising that our results suggest that femoral neck BMD is the dominant risk factor in this patient population with its restricted age range. Ioannidis and colleagues^(^ ^16^ ^)^ used the same tree‐based approach to predict 1‐year fracture risk in a large Canadian long‐term care cohort. Due to the size of the cohort, the terminal nodes for the resulting tree revealed a wide spectrum of short‐term hip fracture risk ranging from <1% to greater than 12.6%. BMD was not measured in the cohort and the major risk factor (ability to walk in the corridors) is likely to be specific to individuals residing in the long‐term care setting. The fracture discrimination in this cohort (C = 0.67) was lower than that in many population‐based cohorts, likely attributable to the lower variation in risk, ie, lower risk gradient and therefore discrimination. Ho‐Le and colleagues^(^ ^40^ ^)^ considered the use of artificial neural nets (ANNs) in a cohort of 1187 older women in the Dubbo cohort of older community‐living Australian adults. They found that ANNs had better sensitivity and specificity at selected cut‐offs for the prediction of hip fracture within 10‐years than other approaches, including logistic regression. Classical approaches rely on statistical testing for the presence of interactions, but it is likely such testing was underpowered with only 54 hip fractures in the derivation cohort. The selected cohort was not limited to the oldest old, and hence age versus BMD interactions may have been important in hip fracture prediction in this population. Thus, in cases where interactions are present in the underlying data a nonparametric approach might be more efficient.

Kruse and colleagues^(^ ^15^ ^)^ used a collection of supervised classification machine learning algorithms (including bagging, boosting, random forests) as well as generalized linear models to determine hip fracture risk from administrative claims data that included BMD testing. Unfortunately, interpretation of the results of this study is challenging. Hip fracture cases and non‐cases had very different mean BMD in unadjusted analysis, but other variables that were similar in cases and controls had higher variable importance. The exceptional performance of the associated model (test area under the curve [AUC] ~0.90) is difficult to explain unless the chosen variables (ie, particular tests including dental consultations and expenditures) were in some way a proxy of clinical diagnosis or treatment of osteoporosis. The black‐box nature of the models in this study limits assessment and interpretation of its findings.

Engels and colleagues^(^ ^14^ ^)^ used a super‐learner algorithm based on combination of machine learning and regression analysis to assess hip fracture risks factors from claims data. The super‐learner model had acceptable performance, but was slightly inferior to that of logistic regression and other component algorithms in the validation set. Thus, although nonparametric approaches may in general be useful, they add a layer of complexity that may not improve model performance. The dominant risk factor for hip fracture is hip BMD, and this risk factor is a robust predictor of hip fracture in parametric and semiparametric models. Hence, the additional flexibility of other modeling approaches may be of limited advantage in hip fracture prediction.

There are many potential advantages to using competing risk random forest models. These advantages include that models are tolerant of highly correlated predictors and there is no need to specify functional form and possible interactions. Although there are algorithms to automate assessment of functional form in classical regression models, these algorithms become challenging when the number of potential predictors increase. The assessment of pairwise interactions is also challenging with a large number of candidate predictors. Random forests leverage the underlying flexibility of tree‐based models and can model nonlinear relationships as well as pairwise and higher‐order interactions. The downside of this flexibility is that there are no equations linking the variables with the estimated risk. Our results show that despite these theoretical benefits, the use of these models for the prediction of hip fracture and mortality before hip fracture does not exceed a simple model that lacks the model flexibility of random forests.

One of the practical implications of the random forest model construction is that there is no way to replicate predictions without an actual forest. Future predictions thus require the original forest (including the original data) or a new forest that replicates the predictions with synthetic data. Model development is also more complex as each data set would generate a different model and there is no easy way to compare model parameters. Hence, validation of prediction models in separate population cohorts is likely to be challenging. It is possible for models with entirely different component variables to result in nearly identical predictions. It is also possible for models with exactly the same component variables to end up with different predictions due to differences due to construction (bootstrap samples, variable choice and cut‐points at each node). Further research is needed to develop appropriate decision rules regarding inclusion of predictors in different contexts. Finally, models created above are not designed to assess causal relationships, ie, the fact that corticosteroids were not selected as an important risk factor for hip fracture risk prediction is not related to causal risk, which should be assessed by different models.

A key limitation of risk prediction or prognostic models is that model performance can never be perfect as they are designed for prediction of future events rather than diagnosis or classification of disease. Results of our random forest competing risk analyses suggest that addition of clinical risk factors yields only a modest improvement for discrimination of future hip fracture events in late‐life adults over that obtained with a models based primarily on total hip and femoral neck BMD. Previous studies in younger participants suggest that including hip structure characteristics that determine bone strength may further improve hip fracture prediction beyond that provided by models based on standard DXA (areal) BMD,^(^ ^41^ ^)^ but incorporating such measures is beyond the scope of the present analysis.

In this study of late‐life adults, we used random forest models accounting for the competing mortality risk to construct prediction models for hip fracture and competing mortality before hip fracture. Our results indicate that although there are overlapping risk factors for hip fracture and competing mortality, hip BMD dominates models predicting hip fracture, while components of phenotypic frailty dominate models predicting death without hip fracture. Advantages of this method included model flexibility and seamless imputation of missing data, but these were counterbalanced by limitations including increasing model complexity and difficulties of model validation. In summary, we found that a standard parsimonious Fine‐Gray model based on major clinical risk factors for hip fracture and mortality may be most appropriate for shared clinical decision‐making regarding whether or not to initiate and continue osteoporosis drug treatment in late‐life adults.

## Author Contributions

**Lisa Langsetmo:** Conceptualization; data curation; formal analysis; funding acquisition; investigation; methodology; writing – original draft; writing – review and editing. **John T Schousboe:** Conceptualization; data curation; funding acquisition; investigation; methodology; writing – original draft; writing – review and editing. **Brent C Taylor:** Investigation; methodology; project administration; writing – review and editing. **Jane A. Cauley:** Funding acquisition; investigation; methodology; writing – review and editing. **Howard A. Fink:** Investigation; methodology; writing – review and editing. **Peggy M. Cawthon:** Data curation; funding acquisition; investigation; methodology; project administration; writing – review and editing. **Deborah M. Kado:** Investigation; methodology; writing – review and editing. **Kristine Ensrud:** Conceptualization; funding acquisition; investigation; methodology; project administration; writing – original draft; writing – review and editing.

## Disclosures

DK reports receiving royalties from UpToDate. The other authors have nothing to disclose.

### Peer Review

The peer review history for this article is available at https://www.webofscience.com/api/gateway/wos/peer-review/10.1002/jbm4.10757.

## Supporting information


**Data S1.** Supporting Information.

## Data Availability

SOF and MrOS data is available to the public via the websites https://sofonline.ucsf.edu and https://mrosonline.ucsf.edu, respectively.
