# Supplementary material for: Advantages and Disadvantages of Random Forest Models for Prediction of Hip Fracture Risk Versus Mortality Risk in the Oldest Old
Source: JBMR Plus. 2023 Jul 3;7(8):e10757. doi: 10.1002/jbm4.10757 (PMC10443071; doi:10.1002/jbm4.10757)
Supplement: Supplementary file 1 — Data S1. Supporting Information. [file JBM4-7-e10757-s001.docx]

**Appendix Table 1. Cohort Study Information**

| Cohort* | Demographics | Geographic Region | Eligibility Criteria | Baseline Exam |
| --- | --- | --- | --- | --- |
| SOF | 9,704 community-dwelling white women, and 662 community-dwelling black women | 4 US sites (Baltimore, MD; Minneapolis, MN; Pittsburgh, PA; Portland, OR) | Women age 65 and older, ability to walk without the assistance of another person, and absence of bilateral hip replacement | 1986-1988 white cohort;  1997-1998 black cohort |
| MrOS | 5,994 community-dwelling men | 6 US sites (Birmingham, AL; Minneapolis, MN; Palo Alto, CA; Pittsburgh, PA; Portland, OR; San Diego, CA) | Men age 65 and older, ability to walk without the assistance of another person, and absence of bilateral hip replacement | 2000-2002 |
| Health ABC | 3,075 black and white community-dwelling men and women | 2 US sites (Memphis, TN and Pittsburgh, PA) | Age 70-79 with no self-report of mobility difficulty or mobility disability | 1997-1998 |

**Appendix Table 2. Study Participants**

|  | Men | | |  | Women | | |
| --- | --- | --- | --- | --- | --- | --- | --- |
| Cohort | Study  Year | Calendar  Year | N | Cohort | Study  Year | Calendar  Year | N |
| MrOS | 4.5 | 2005-2007 | 1512 | SOF | 10 | 1996-1998 | 2311 |
|  | 7 | 2007-2009 | 526 |  | 16 | 2002-2004 | 1820 |
|  | 14 | 2014-2016 | 1167 |  |  |  |  |
| HABC | 3 | 1999-2000 | 149 | HABC | 3 | 1999-2000 | 145 |
|  | 5/6 | 2001-2003 | 188 |  | 5/6 | 2001-2003 | 177 |
|  | 8 | 2004-2005 | 252 |  | 8 | 2004-2005 | 268 |
|  | 10 | 2006-2007 | 195 |  | 10 | 2006-2007 | 232 |
| Combined | |  | 3989 |  | |  | 4953 |

**Appendix Table 3. Variable Ranking by Minimal Depth (MD) and Variable Importance (VIMP) for Hip Fracture and Mortality before Hip Fracture among Men and Women**

|  | Men | | | Women | | |
| --- | --- | --- | --- | --- | --- | --- |
| Variable | **MD Rank** | Fracture Rank  (VIMP) | Mortality Rank (VIMP) | **MD Rank** | Fracture Rank (VIMP) | Mortality Rank (VIMP) |
| Total hip BMD | 8 | 2 |  | 1 | 2 | 7 |
| Gait speed | 2 | 8 | 5 | 2 | 4 | 1 |
| Weight change since age 25y | 3 |  | 15 | 3 | 3 | 2 |
| Femoral neck BMD | 11 | 1 |  | 4 | 1 | 20 |
| Age | 7 | 15 | 6 | 5 |  | 3 |
| Weight | 6 |  | 17 | 6 | 5 | 6 |
| # Frailty components | 1 |  | 1 | 7 | 8 | 10 |
| Chair stand speed | 4 | 4 | 8 | 8 |  | 11 |
| Mobility score | 9 | 6 | 12 | 9 |  | 8 |
| Grip strength | 5 |  | 3 | 10 |  |  |
| # Chronic conditions | 10 | 13 | 16 | 11 | 11 | 16 |
| # Medications | 15 | 5 |  | 12 |  |  |
| Height loss since age 25y | 19 |  | 20 | 13 |  | 18 |
| Difficulty walking 3 blocks | 16 | 9 | 9 | 14 | 9 | 5 |
| Height | 18 |  |  | 15 | 14 |  |
| Health status | 12 |  | 18 | 16 |  | 14 |
| Falls history (12m) | 22 | 7 |  | 17 |  |  |
| Fracture history | 26 | 14 |  | 18 | 7 |  |
| Shrinking (weight loss) | 17 |  | 7 | 19 |  | 15 |
| Slow gait | 14 | 11 | 2 | 20 | 6 |  |
| Difficulty climbing 10 steps | 24 | 12 |  | 21 |  |  |
| Weakness (chair stand) | 21 | 3 | 4 | 22 | 10 | 4 |
| Loop diuretics | 20 |  | 13 | 23 |  | 12 |
| Dementia | 13 | 10 | 10 | 24 | 12 |  |
| Heart disease |  |  |  | 25 |  | 9 |
| Warfarin | 25 |  |  | 26 |  | 13 |
| SSRIs |  |  |  | 27 |  | 19 |
| Stroke | 31 |  |  | 28 |  |  |
| Corticosteroids | 27 |  |  | 29 |  |  |
| Diabetes |  |  |  | 30 |  |  |
| Heart failure | 23 |  | 14 | 31 |  | 17 |
| Arthritis |  |  |  | 32 |  |  |
| Smoking history | 32 |  | 19 | 33 |  |  |
| Beta blockers |  |  |  | 34 |  |  |
| Benzodiazepines | 28 |  |  | 35 |  |  |
| Weakness (grip strength) | 33 |  | 11 | 36 |  |  |
| Cancer |  |  |  | 37 |  |  |
| Parkinsonism | 29 |  |  |  |  |  |
| Low physical activity | 30 |  |  |  |  |  |
| H1 receptor antagonists | 34 |  |  |  |  |  |
| COPD |  |  |  |  | 13 |  |

**Minimal depth (MD) obtained from model predicting both outcomes, variable importance (VIMP) obtained from outcome specific model. Variables with MD > threshold [10 (men), 9.5 (women)] or VIMP<0.0015 not ranked

**Code Sample and R-Markdown Output.**

R version 4.2.2 (2022-10-31 ucrt) -- "Innocent and Trusting"

Copyright (C) 2022 The R Foundation for Statistical Computing

Platform: x86_64-w64-mingw32/x64 (64-bit)

randomForestSRC, version 3.1.1

ggRandomForest, version 2.2.1

####Run large model predicting both hip fracture and mortality

set.seed(12345) # Set seed for reproducibility

forest_lr_all <- rfsrc((f),

data=impute_data0,

splitrule = "logrankCR",

ntree = 1000,

nsplit = 10,

importance = "permute",

ensemble = "oob",

ntime = 50,

forest = TRUE,

var.used = "all.trees",

tree.err = TRUE,

terminal.qualts = TRUE,

predicted = TRUE,

predicted.oob = TRUE,

survival.oob = TRUE,

inbag = TRUE)

####Determine minimal depth of variables from the above model

md_fx_all <- gg_minimal_depth(forest_lr_all)

md_var <- paste(md_fx_all$topvars, collapse=" + ")

md_formula <- as.formula(paste(svar, md_var))

####Rerun model with only MD selected variables

set.seed(12345) # Set seed for reproducibility

forest_md <- rfsrc((md_formula),

data=impute_data0,

splitrule = "logrankCR",

ntree = 1000,

nsplit = 10,

importance = "permute",

ensemble = "oob",

ntime = 50,

forest = TRUE,

var.used = "all.trees",

tree.err = TRUE,

terminal.qualts = TRUE,

predicted = TRUE,

predicted.oob = TRUE,

survival.oob = TRUE,

inbag = TRUE)

######Run large model predicting hip fracture outcome

set.seed(12345)

forest_fx_all <- rfsrc((f),

data=impute_data1,

splitrule = "logrank",

cause = c(1,0),

ntree = 1000,

nsplit = 10,

importance = "permute",

ensemble = "oob",

ntime = 50,

forest = TRUE,

var.used = "all.trees",

tree.err = TRUE,

terminal.qualts = TRUE,

predicted = TRUE,

predicted.oob = TRUE,

survival.oob = TRUE,

inbag = TRUE)

set.seed(12345)

vimp_fx_all <- gg_vimp(forest_fx_all, importance="permute")

df_fx_all <- as.data.frame(vimp_fx_all)

filter_fx2 <- df_fx_all %>% filter(set=="event.1"&positive==TRUE& vimp>0.002)

newvar_fx2 <- as.vector(filter_fx2$vars)

avsfx2 <- paste(newvar_fx2, collapse=" + ")

fxl2 <- as.formula(paste(svar, avsfx2))

filter_fx3 <- df_fx_all %>% filter(set=="event.1"&positive==TRUE& vimp>0.003)

newvar_fx3 <- as.vector(filter_fx3$vars)

avsfx3 <- paste(newvar_fx3, collapse=" + ")

fxl3 <- as.formula(paste(svar, avsfx3))

#####Re-run model using VIMP selected variables

set.seed(12345)

forest_fx_vimp2 <- rfsrc((fxl2),

data=impute_data1,

splitrule = "logrank",

cause = c(1,0),

ntree = 1000,

nsplit = 10,

importance = "permute",

ensemble = "oob",

ntime = 50,

forest = TRUE,

var.used = "all.trees",

tree.err = TRUE,

terminal.qualts = TRUE,

predicted = TRUE,

predicted.oob = TRUE,

survival.oob = TRUE, inbag = TRUE)

set.seed(12345) # Set seed for reproducibility

forest_fx_vimp3 <- rfsrc((fxl3),

data=impute_data1,

splitrule = "logrank",

cause = c(1,0),

ntree = 1000,

nsplit = 10,

importance = "permute",

ensemble = "oob",

ntime = 50,

forest = TRUE,

var.used = "all.trees",

tree.err = TRUE,

terminal.qualts = TRUE,

predicted = TRUE,

predicted.oob = TRUE,

survival.oob = TRUE,

inbag = TRUE)

###Similar code for mortality

Hip fracture in women, single pass VIMP (permute)

Langsetmo

2023-02-09

####Select variables using minimal depth

## Sample size: 4953
## Number of events: 325, 824
## Number of trees: 1000
## Forest terminal node size: 15
## Average no. of terminal nodes: 228.073
## No. of variables tried at each split: 8
## Total no. of variables: 59
## Resampling used to grow trees: swor
## Resample size used to grow trees: 3130
## Analysis: RSF
## Family: surv-CR
## Splitting rule: logrankCR *random*
## Number of random split points: 10
## (OOB) Requested performance error: 0.25778247, 0.32433585

## minimal depth variable selection ...
##
##
## -----------------------------------------------------------
## family : surv-CR
## var. selection : Minimal Depth
## conservativeness : medium
## x-weighting used? : TRUE
## dimension : 59
## sample size : 4953
## ntree : 1000
## nsplit : 10
## mtry : 8
## nodesize : 15
## refitted forest : FALSE
## model size : 36
## depth threshold : 9.5504
## PE (true OOB) : 25.7782 32.4336
##
##
## Top variables:
## depth vimp.event.1 vimp.event.2
## bmd 2.492 0.041 0.006
## walk_sp 2.731 0.009 0.012
## perc_age25weight 2.935 0.009 0.008
## fnbmd 2.954 0.037 0.002
## age 3.358 0.004 0.007
## weight 3.415 0.005 0.006
## frailty_comp_impute 3.701 0.003 0.004
## chair1s 3.850 0.002 0.005
## mobility 4.208 0.001 0.004
## grip_st 4.330 0.002 0.001
## meds 4.654 0.001 0.000
## index_cat 4.698 0.002 0.002
## hl_cat 4.726 0.000 0.002
## iadl_walk 4.881 0.003 0.004
## height 5.238 0.001 0.000
## health_stat 5.396 0.000 0.002
## fall_cat 5.671 0.000 0.002
## prior_fx2 5.973 0.003 0.000
## weak_chair 6.018 0.003 0.006
## slow 6.054 0.006 0.002
## iadl_step 6.180 0.000 0.002
## dementia2 6.286 0.001 0.001
## shrinking 6.381 0.001 0.002
## loopd 6.509 0.000 0.002
## warfarin 6.664 0.000 0.002
## chd 7.230 0.000 0.002
## ssri 7.250 0.000 0.002
## stroke 7.681 0.000 0.001
## cortico 8.083 -0.001 0.000
## chf 8.286 0.000 0.001
## diabetes2 8.555 0.000 0.000
## betab 8.944 0.000 0.001
## arthritis 8.983 0.000 0.001
## cancer 9.100 0.001 0.001
## ever_smoke 9.207 -0.001 0.000
## benzo 9.379 0.000 0.000
## -----------------------------------------------------------

## Surv(time, status) ~ bmd + walk_sp + perc_age25weight + fnbmd +
## age + weight + frailty_comp_impute + chair1s + mobility +
## grip_st + meds + index_cat + hl_cat + iadl_walk + height +
## health_stat + fall_cat + prior_fx2 + weak_chair + slow +
## iadl_step + dementia2 + shrinking + loopd + warfarin + chd +
## ssri + stroke + cortico + chf + diabetes2 + betab + arthritis +
## cancer + ever_smoke + benzo

## Sample size: 4953
## Number of events: 325, 824
## Number of trees: 1000
## Forest terminal node size: 15
## Average no. of terminal nodes: 223.6
## No. of variables tried at each split: 6
## Total no. of variables: 36
## Resampling used to grow trees: swor
## Resample size used to grow trees: 3130
## Analysis: RSF
## Family: surv-CR
## Splitting rule: logrankCR *random*
## Number of random split points: 10
## (OOB) Requested performance error: 0.25745111, 0.32304673

###Make forests for fracture using VIMP as selection criteria. Run all variables.

## Sample size: 4953
## Number of events: 325, 824
## Number of trees: 1000
## Forest terminal node size: 15
## Average no. of terminal nodes: 264.481
## No. of variables tried at each split: 8
## Total no. of variables: 59
## Resampling used to grow trees: swor
## Resample size used to grow trees: 3130
## Analysis: RSF
## Family: surv-CR
## Splitting rule: logrank *random*
## Number of random split points: 10
## (OOB) Requested performance error: 0.26081175, 0.3251333

###Select variables by VIMP

###Larger fracture forest (VIMP>0.002)

## Surv(time, status) ~ fnbmd + bmd + perc_age25weight + walk_sp +
## prior_fx2 + weight + slow + age + height + weak_chair + iadl_walk

## Sample size: 4953
## Number of events: 325, 824
## Number of trees: 1000
## Forest terminal node size: 15
## Average no. of terminal nodes: 287.666
## No. of variables tried at each split: 4
## Total no. of variables: 11
## Resampling used to grow trees: swor
## Resample size used to grow trees: 3130
## Analysis: RSF
## Family: surv-CR
## Splitting rule: logrank *random*
## Number of random split points: 10
## (OOB) Requested performance error: 0.25891437, 0.34371169

###Parsimonious fracture forest (VIMP>0.003)

## Surv(time, status) ~ fnbmd + bmd + perc_age25weight + walk_sp +
## prior_fx2 + weight + slow

## Sample size: 4953
## Number of events: 325, 824
## Number of trees: 1000
## Forest terminal node size: 15
## Average no. of terminal nodes: 293.207
## No. of variables tried at each split: 3
## Total no. of variables: 7
## Resampling used to grow trees: swor
## Resample size used to grow trees: 3130
## Analysis: RSF
## Family: surv-CR
## Splitting rule: logrank *random*
## Number of random split points: 10
## (OOB) Requested performance error: 0.26939296, 0.35110274

###Make forests for mortality using VIMP as selection criteria. Run all variables.

## Sample size: 4953
## Number of events: 325, 824
## Number of trees: 1000
## Forest terminal node size: 15
## Average no. of terminal nodes: 257.135
## No. of variables tried at each split: 8
## Total no. of variables: 59
## Resampling used to grow trees: swor
## Resample size used to grow trees: 3130
## Analysis: RSF
## Family: surv-CR
## Splitting rule: logrank *random*
## Number of random split points: 10
## (OOB) Requested performance error: 0.26715307, 0.32612138

###Select variables for mortality

###Larger mortality forest (VIMP>0.002)

## Surv(time, status) ~ walk_sp + perc_age25weight + age + weak_chair +
## weight + iadl_walk + bmd + mobility + chair1s + frailty_comp_impute +
## chd + loopd + index_cat + warfarin + health_stat

## Sample size: 4953
## Number of events: 325, 824
## Number of trees: 1000
## Forest terminal node size: 15
## Average no. of terminal nodes: 242.927
## No. of variables tried at each split: 4
## Total no. of variables: 15
## Resampling used to grow trees: swor
## Resample size used to grow trees: 3130
## Analysis: RSF
## Family: surv-CR
## Splitting rule: logrank *random*
## Number of random split points: 10
## (OOB) Requested performance error: 0.27369193, 0.32474938

###Parsimonious mortality forest

## Surv(time, status) ~ walk_sp + perc_age25weight + age + weak_chair +
## weight + iadl_walk + bmd + mobility + chair1s + frailty_comp_impute +
## chd + loopd

## Sample size: 4953
## Number of events: 325, 824
## Number of trees: 1000
## Forest terminal node size: 15
## Average no. of terminal nodes: 247.778
## No. of variables tried at each split: 4
## Total no. of variables: 12
## Resampling used to grow trees: swor
## Resample size used to grow trees: 3130
## Analysis: RSF
## Family: surv-CR
## Splitting rule: logrank *random*
## Number of random split points: 10
## (OOB) Requested performance error: 0.27053043, 0.3315632
